# A Fault Feature Extraction Method Based on Improved VMD Multi-Scale Dispersion Entropy and TVD-CYCBD

**DOI:** 10.3390/e25020277

**Published:** 2023-02-02

**Authors:** Jingzong Yang, Chengjiang Zhou, Xuefeng Li, Anning Pan, Tianqing Yang

**Affiliations:** 1 School of Dig Data, Baoshan University, Baoshan 678000, China; 2 School of Information, Yunnan Normal University, Kunming 650500, China; 3 College of Automobile and Traffic Engineering, Nanjing Forestry University, Nanjing 210037, China

**Keywords:** rolling bearing, fault feature extraction, VMD, TVD, CYCBD

## Abstract

In modern industry, due to the poor working environment and the complex working conditions of mechanical equipment, the characteristics of the impact signals caused by faults are often submerged in strong background signals and noises. Therefore, it is difficult to effectivelyextract the fault features. In this paper, a fault feature extraction method based on improved VMD multi-scale dispersion entropy and TVD-CYCBD is proposed. First, the marine predator algorithm (MPA) is used to optimize the modal components and penalty factors in VMD. Second, the optimized VMD is used to model and decompose the fault signal, and then the optimal signal components are filtered according to the combined weight index criteria. Third, TVD is used to denoise the optimal signal components. Finally, CYCBD filters the de-noised signal and then envelope demodulation analysis is carried out. Through the simulation signal experiment and the actual fault signal experiment, the results verified that multiple frequency doubling peaks can be seen from the envelope spectrum, and there is little interference near the peak, which shows the good performance of the method.

## 1. Introduction

With the continuous acceleration of the industrialization process in the world, modern industry is gradually developing towards the direction of large-scale, complex, high-speed, and automatic production equipment. Especially since the concept of Industry 4.0 was presented, the state data generated by the operation of mechanical equipment has increased [1,2]. As the most basic and essential mechanical components, rolling bearings are widely used in modern aviation, aerospace, navigation, machine tools, etc. Rolling bearing runs under poor working conditions for a long time, so it is easy to have various faults. It may cause excessive energy consumption, cause a bad mechanical equipment vibration, and affect the performance of the relevant instruments on the equipment. In the worst case, it will lead to the shutdown of mechanical equipment, causing substantial economic losses and even endangering the life safety of the relevant staff. Therefore, the fault diagnosis of this component can not only ensure the smooth and healthy operation of mechanical equipment but also help prevent major accidents. Generally, due to the limitation of the working environment of bearing, it is impossible to diagnose directly. In the process of a practical application, the vibration signal extracted by observing the running state of the bearing contains a lot of information. Meanwhile, the requirements for equipment and staff skills are low in the process of signal acquisition. Therefore, vibration signal analysis has become one of the most widelyused fault detection methods. Because the bearingvibration signal under fault conditions shows prominent non-stationary, nonlinear, and weak fault characteristics [3], it is important and difficult for experts and scholars in this field to enhance fault information from the noise background.

As an effective method, time-frequency analysis has been widely used in the processing of nonlinear and non-stationary signals of mechanical equipment. So far, many experts and scholars have proposed various time-frequency analysis methods. Traditional methods include short-time Fourier transform (SFFT) [4,5], Wigner Ville distribution [6,7], wavelet transform [8,9], etc. The above methods have achieved good results in practical applications. Burriel Valencia J et al. [10] used SFFT to diagnose faults in induction motors operating under transient conditions. The results show that it is suitable for online diagnosis. Wu J D et al. [11] developed an engine platform diagnostic system. The system uses the Wigner Ville distribution to extract the instantaneous capability map as a feature quantity and input it into the upgraded network model for modeling. The experimental results show that the system has achieved good results. KankarP K et al. [12] extracted the feature quantity from the wavelet coefficients of the collected vibration signal and performed the fault diagnosis in the local defects of the bearing components. The results of the fault classification show that the accuracy of the fault identification using the support vector machine is higher. However, there are still some defects in the use of these methods. SFFT has a low time-frequency resolution. Meanwhile, the short-time SFFT cannot meet the two conditions of the resolution of the time and frequency domain. The variation adopted by the Wigner Ville distribution is nonlinear and cross-interference will occur when the multi-component analysis is carried out. The decomposition of multi-component mixed signals by a wavelet transform will lead to the problem of mode aliasing. At the same time, there is no reference standard for selecting appropriate wavelet bases. In addition, the above method cannot adaptively decompose the signal.

Compared with the traditional methods above, time-frequency domain analysis methods such as empirical mode decomposition (EMD), cyclic symplectic component decomposition (CSCD), symplectic geometry packet decomposition (SGPD), and ensemble empirical mode decomposition (EEMD) proposed by Huang and other scholars [13,14,15,16] can adaptively decompose signals. Therefore, it shows certain advantages. Sun Y et al. [17] introduced the EMD and improved Chebyshev distance into the bearing fault diagnosis. Experiments show that the method can diagnose bearing faults by using the signal components obtained from EMD to construct the improved Chebyshev distance. Cui H et al. [18] introduced EMD in the fault diagnosis of weak signals. The results show that it can improve the quality of decomposition. Aiming at the intermittent faults in analog circuits, Zhong T et al. [19] offered a method combining EEMD and a deep belief network (DBN). They concluded that the method can independently select features and diagnosis intermittent faults and has a higher fault diagnosis accuracy than other standard methods. In an attempt to solve the problem of many harmonic components and noise signals mixed in the signals under complex operating conditions, Wang L et al. [20] introduced a method combining EEMD and improved sparse representation. The experimental results show that it can effectively extract the shock components from the signals. Although EMD and EEMD have been tested in practical applications, the idea of this method is to follow the recursive decomposition pattern. Therefore, there are usually problems with mode aliasing and the end effect. In recent years, Dragomiretskiy et al. [21] developed another method, namely, variational mode decomposition (VMD). As a signal processing method, this method decomposes the signal through non-recursive and variational modal decomposition mode to overcome modal aliasing and the endpoint effect and has been applied in fault diagnosis. Aiming at the diagnosis problem of bearing weak fault signals caused by long transmission paths, Cui H et al. [22] introduced an algorithm combining VMD and maximum correlated kurtosis deconvolution (MCKD). The results show that it can diagnose rolling element faults. Ye M et al. [23] introduced VMD in the diagnosis of bearing fault. First, the original signal is decomposed into multiple signal components. Then, multi-scale replacement entropy is extracted from the original signal to complete the modeling of the pattern recognition model. Experiments verify the effectiveness of this method. Since the early fault signal characteristics of hydro generators are weak, it is difficult to extract the fault characteristics. Tang X et al. [24] used the technique of combining VMD and a singular value for fault diagnosis. The method’s accuracy is verified by analyzing the vibration data of the actual hydropower station. To effectively identify the early fault characteristics of the gearbox, Mansi, Saini K et al. [25] introduced an algorithm by combining the maximum overlap discrete wavelet transform and the VMD. By comparing the recognition effects of the different classifiers, it is concluded that the fault features extracted by VMD are more conducive to accurately dividing the fault stages.

However, there is still some noise in the signal components after decomposition by time-frequency analysis of the rolling bearing fault signal under complex working conditions. If fault analysis is conducted directly, the noise interference will harm the development of the research work. To effectively reduce noise interference, Rudin et al. [26] proposed the total variation denoising algorithm (TVD) and applied it to reduce the image noise. This method has achieved good results in the application process. TVD not only retains significant edges but also enhances the image’s structure. At present, the algorithm has been applied in many aspects of research work. Kumar S S et al. [27] proposed a TVD-based ECG R-peak location algorithm. The experiment provedthat the algorithm can effectively retain the signal’s steep slope or peak value and has a high accuracy. Wan Z et al. [28] proposed a kurtosis-wavelet total variation denoising model. The experiments verified the effectiveness and robustness of this method. Lv D et al. [29] proposed an improved TVD algorithm. The results show that the algorithm can effectively improve the denoising effect.

In this paper, a method based on improved VMD multi-scale dispersion entropy and total variation denoising(TVD)-maximum second-order cyclostationary blind convolution (CYCBD) is proposed.First, according to the optimization principle of minimizing the mean value of dispersion entropy, the marine predator algorithm (MPA) is used to optimize the modal components and penalty factors in VMD to optimize the initialization parameters. Second, the optimization results are input into VMD for modeling and the fault signals are decomposed adaptively. Then, according to the combined weight screening criteria which were constructed, the optimal signal component is selected for TVD noise reduction and the noise-reduced signal is input into the CYCBD algorithm for filtering to further enhance the shock characteristics in the signal. Finally, the envelope spectrum of the signal is analyzed to extract the fault characteristic frequency.

The main contributions of this study are as follows:

(1) The strategy for optimizing VMD by MPA is proposed, and the initialization parameters of the algorithm are optimized. It enables the signal to be decomposed with a high quality and eliminates adverse effects, such as mode aliasing.
(2) A combined weight screening criterion that balances the advantages and disadvantages of the two indicators is constructed. On the basis of this, the evaluation of the IMF signal components and the selection of the best signal components are completed. Meanwhile, the signal is subsequently processed by TVD noise reduction to reduce the noise interference.
(3) After CYCBD filtering, the periodic pulse characteristics of the fault signals can be effectively enhanced. It makes the extracted bearing fault feature frequency clear and richer. It can provide an important referencemethod for solving the problem of fault feature extraction of rolling bearings.

The arrangement of this paper is as follows: Section 2 describes the basic theory of improved VMD, multi-scale dispersion entropy, TVD, and CYCBD algorithms. Section 3 introduces the specific steps and flow chart of the improved VMD multi-scale dispersion entropy and TVD-CYCBD. Section 4 validates the feasibility of the proposed method. Section 5 shows the practical application performance of the proposed method. Section 6 draws a conclusion.

## 2. Theoretical Background

### 2.1. Improved VMD

The main parameters of the VMD algorithminclude the modal components and penalty factors. The modal component ensures the appropriateness and accuracy of the number of decomposition modes. The penalty factor is related to the accuracy of the signal reconstruction. The selection of the two factors plays an essential role in the decomposition effect of the VMD. When the modal component and penalty factor are too large, it is easy to cause modal aliasing. Otherwise, it will cause the loss of useful information. The marine predator algorithm (MPA) proposed in recent years has the advantages of a simple structure, flexible algorithm, easy implementation, and the ability to coordinate exploration and development. Compared with other algorithms, it has a better optimization performance. Therefore, this paper introduced MPA to optimize VMD. Then, the optimization results are entered into VMD for modeling.

#### 2.1.1. VMD

VMD is an adaptive signal processing method proposed by Dragomiretskiy. It decomposes the signal into finite component signals. The component signal is the limited bandwidth of a specific center frequency, which satisfies that the sum of all the component signals is equal to the original signal. The signal is solved iteratively to minimize the sum of the bandwidth. It is defined as follows:

(1) $$\mu_{k}(t)=A_{k}(t)\cos[\phi_{k}(t)]$$

where $A_{k}(t)$ is the instantaneous amplitude. $\phi_{k}(t)$ is a phase. $\omega_{k}(t)$ is the instantaneous phase.

(2) $$\omega_{k}(t)=\phi_{k}{}^{\prime }(t)=\frac{d\phi_{k}(t)}{dt}$$

By decomposing the original signal, discrete modes $\mu_{k}(t)\;(k\in 1,2,\cdot \cdot \cdot ,k)$ can be obtained. Then, it follows the following process.

(1) The unilateral spectrum of each modal component is calculated by Hilbert.

(3) $$\left(\delta (t)+\frac{j}{\pi t})*\mu_{k}(t\right)$$

where $\mu_{k}(t)$ is the *k*-th modal. δ(t) is the unit pulse function. ***j*** is an imaginary number.

(2) The center frequency is estimated and the spectrum of each mode is modulated to the corresponding fundamental frequency band by exponential correction $e_{}^{-j\omega_{k}t}$.

(4) $$[(\delta (t)+\frac{j}{\pi t})*\mu_{k}(t)]e_{}^{-j\omega_{k}t}$$

(3) Find the square L^2^ norm of the demodulation signal gradient.

(5) $${\left\|d_{t}[(\delta (t)+\frac{j}{\pi t})*\mu_{k}(t)]e_{}^{-j\omega_{k}t}\right\|}_{2}^{2}$$

The constraint model is built on the basis of the above formula, and then:

(6) $$\left\{\begin{array}{l} \underset{\left\{\mu_{k}\right\},\left\{\omega_{k}\right\}}{\min}\left\{\sum_{k}^{}{\left\|d_{t}[(\delta (t)+\frac{j}{\pi t})*\mu_{k}(t)]e_{}^{-j\omega_{k}t}\right\|}_{2}^{2}\right\}\\ s.t.\;\;\sum_{k}\mu_{k}(t)=x(t) \end{array}\right.$$

where $\left\{\omega_{k}\right\}=\left\{\omega_{1},\omega_{2},\omega_{3},\cdot \cdot \cdot \omega_{k}\right\}$ is the *k*-th central frequency of the signal after VMD. *t* is the time. δ(t) is the Dirac function. *j* is an imaginary unit. ∗ is a convolution operation. ${\left\|\right\|}_{2}^{2}$ is the L2 norm. *x*(*t*) is the original signal.

To calculate the above model, the penalty factor and Lagrangian multiplication operator are introduced in the above equation. Then, the problem to be solved is transformed into an unconstrained variational problem and the following formula can be obtained:

(7) $$L(\left\{\mu_{k}\right\},\left\{\omega_{k}\right\},\lambda )=\alpha {\sum_{k}{\left\|d_{t}[(\delta (t)+\frac{j}{\pi t})*\mu_{k}(t)]e_{}^{-j\omega_{k}t}\right\|}_{2}^{2}+\left\|x(t)\;-\sum_{k}\mu_{k}(t)\right\|}_{2}^{2}+\langle \lambda (t),x(t)-\sum_{k}\mu_{k}(t)\rangle$$

#### 2.1.2. MPA Method

The main inspiration for MPA [30] comes from the foraging strategy of marine predators, the Lévy motion and Brownian motion, and the optimal encounter strategy of the interaction between predators and prey. The mathematical description of the MPA optimization process is as follows:

(1) Initialization phase. To commence the optimization process, the MPA algorithm will randomly initialize the prey location in the search space. The expression formula is as follows:

(8) $$X_{0}=X_{\min}+rand(X_{\max}-X_{\min})$$

where $X_{\max}$ and $X_{\min}$ are the upper and lower bounds of the search space.

(2) MPA optimization stage. At the initial stage, under the condition of a high-speed ratio, the predator does not move. When $Iter_{}<\frac{1}{3}Max\_Iter$, the mathematical description of the MPA optimization process is as follows:

(9) $$\begin{array}{l} \left\{\begin{array}{l} stepsice_{i}=R_{B}⊗(Elite_{i}-R_{B}⊗Prey_{i})\\ Prey_{i}=Prey_{i}+P●R⊗stepsice_{i} \end{array}\right.\;\;\;i=1,2,\ldots ,n;\\ \;\;Iter_{}<\frac{1}{3}Max\_Iter \end{array}$$

where stepsice is the moving step. $Elite_{i}$ is the elite matrix. $Prey_{i}$ is the prey matrix. *P* is a constant. *P* is taken as 0.5. *R* and is the uniform random vector. $R_{B}$ is the random vector. *Iter*, *Max*_*Iter* is the current iteration number and the maximum iteration number.

In the middle of the iteration, the optimization process is transformed from the exploration to the development when the speed of the predator and prey is the same. Therefore, half will be used for exploration and the other half for development. The mathematical descriptions of the development and exploration are as follows:

(10) $$\begin{array}{l} \left\{\begin{array}{l} stepsice_{i}=R_{L}⊗(Elite_{i}-R_{L}⊗Prey_{i})\\ Prey_{i}=Prey_{i}+P●R⊗stepsice_{i} \end{array}\right.\;\;\;i=1,2,\ldots ,n/2;\\ \;\;\frac{1}{3}Max\_Iter<Iter_{}<\frac{2}{3}Max\_Iter \end{array}$$

(11) $$\begin{array}{l} \left\{\begin{array}{l} stepsice_{i}=R_{B}⊗(R_{B}⊗Elite_{i}-Prey_{i})\\ Prey_{i}=Elite_{i}+P\;●\;CF⊗stepsice_{i} \end{array}\right.\;\;\;i=n/2,\ldots ,n;\\ \;\;\frac{1}{3}Max\_Iter<Iter_{}<\frac{2}{3}Max\_Iter \end{array}$$

where $R_{L}$ is a random vector with Lévy distribution. *CF* = (1 − *Iter*/*Max*_*Iter*)^(2·*Iter*/*Max*_*Iter*)^.

At the end of the iteration, the predator moves based on the way of Lévy walking and its position changes as follows:

(12) $$\begin{array}{l} \left\{\begin{array}{l} stepsice_{i}=R_{L}⊗(R_{L}⊗Elite_{i}-Prey_{i})\\ Prey_{i}=Elite_{i}+P\;●\;CF⊗stepsice_{i} \end{array}\right.\;\;\;i=1,2,\ldots ,n;\\ \;\;Iter_{}>\frac{2}{3}Max\_Iter \end{array}$$

(3) Fish aggregating devices (*FADs*) effect and eddy the current effect. This strategy can enable MPA to overcome the premature convergence problem in the optimization process and escape from the local extremum.

### 2.2. Total Variation Denoising

TVD [24] is a nonlinear fast noise reduction method without iteration, which achieves the smooth processing of discrete signals.

Suppose that the signal *x* with *N* points is shown as follows:

(13) $$x=[x_{0},x_{1},x_{2},\ldots ,x_{N-1}]$$

The first-order differential of the matrix (*N* − 1 × *N*) is as follows:

(14) $$D_{1}=\left[\begin{array}{l} -1\;\;\;\;\;1\;\;\;\;\;\;\\ \;\;\;\;\;\;-1\;\;\;\;\;1\;\;\\ \;\;\;\;\;\;\;\;\;\;\;\;\;\;\;\vdots \;\;\;\;\;\vdots \;\;\;\\ \;\;\;\;\;\;\;\;\;\;\;\;\;\;\;\;\;\;-1\;\;\;\;\;1\;\;\; \end{array}\right]$$

The second-order differential of the matrix (*N* − 2 ×*N*) is as follows:

(15) $$D_{2}=\left[\begin{array}{l} -1\;\;\;\;2\;\;\;\;-1\;\;\;\;\;\;\\ \;\;\;\;\;-1\;\;\;\;\;\;\;2\;\;\;\;-1\;\\ \;\;\;\;\;\;\;\;\;\;\;\;\;\;\;\;\vdots \;\;\;\;\;\;\;\;\vdots \;\;\;\;\;\;\;\vdots \;\;\;\\ \;\;\;\;\;\;\;\;\;\;\;\;\;\;\;\;\;\;\;\;\;-1\;\;\;\;\;\;\;2\;\;\;\;-1\; \end{array}\right]$$

The expression of the $l_{p}$ norm (P≥1) is as follows:

(16) $${\left\|x\right\|}_{p}={\left({\left\|x_{1}\right\|}_{}^{p}+{\left\|x_{2}\right\|}_{}^{p}\;+\cdots +{\left\|x_{N}\right\|}_{}^{p}\right)}_{}^{\frac{1}{p}}$$

In special cases, when *p* = 1, Equation (16) will become the following form:

(17) $${\left\|x\right\|}_{1}=(\left|x_{1}\right|+\left|x_{2}\right|+\cdots +\left|x_{N}\right|)$$

When *p* = 2, Equation (16) becomes the following form:

(18) $$\mathrm{TV}(x)={\left\|D_{1}x\right\|}_{1}=\sum_{n=1}^{N-1}\left|x(n)-x(n-1)\right|$$

Assume that the signal *y*(*n*) containing noise is:

(19) y(x)=x(n)+w(n)    (n=0,1,…,N−1)

The noise reduction of this algorithm can be summarized into the following optimization problems:

(20) $$\underset{}{\underset{x}{\arg\min}{\left\|y-x\right\|}_{2}^{2}}+\lambda {\left|D_{1}x\right|}_{1}$$

### 2.3. CYCBD Method

The CYCBD maximizes the second-order cyclostationarity (ICS_2_) by solving the eigenvalue to extract the required fault signal from the complex source signal. The convolution process is expressed as follows:

(21) s=x⊗h

where *s* is the original signal. *x* is the observation signal. *h* is the inverse filter.

The above formula can be expressed as follows:

(22) s=Xh

(23) $$\left[\begin{array}{l} s[N-1]\\ \;\;\;\;\;\;\vdots \\ s[L-1] \end{array}\right]=\left[\begin{array}{l} x[N-1]\;\;\;\cdots \;\;\;\;\;\;\;\;\;x[0]\\ \;\;\;\;\;\vdots \;\;\;\;\;\;\;\;\;\;\vdots \;\;\;\;\;\;\;\;\;\;\;\;\;\;\vdots \\ x[L-1]\;\;\;\;\cdots \;\;\;\;x[L-N-2] \end{array}\right]\;●\;\left[\begin{array}{l} \;\;\;h[0]\\ \;\;\;\;\;\;\vdots \\ h[N-1] \end{array}\right]\;$$

where *L* is the length of discrete signal *s*. *N* is the length of the inverse filter *h*.

Therefore, *ICS*_2_ can be expressed in the form of generalized Rayleigh entropy:

(24) $$ICS_{2}=\frac{h_{}^{H}X_{}^{H}WXh}{h_{}^{H}X_{}^{H}Xh}=\frac{h_{}^{H}R_{XWX}h}{h_{}^{H}R_{XX}h}$$

where *H* is the conjugate transpose of the matrix. $R_{XWX}$ is the weighted correlation matrix. $R_{XX}$ is the correlation matrix. The weighting matrix *W* is as follows.

(25) $$W=diag(\frac{p[{\left|s\right|}_{}^{2}]}{s_{}^{H}s})(L-N+1)$$

(26) $$p[{\left|s\right|}_{}^{2}]=\frac{EE_{}^{H}{\left|s\right|}_{}^{2}}{L-N+1}$$

(27) $${\left|s\right|}_{}^{2}=[s{\left|N-1\right|}_{}^{2},\ldots ,\;s{\left|L-1\right|}_{}^{2}{]}_{}^{T}$$

(28) $$E=\left[\begin{array}{l} e_{}^{-j2\pi \frac{1}{T}(N-1)}\;\;\cdots \;\;e_{}^{-j2\pi \frac{K}{T}(N-1)}\\ \;\;\;\;\;\;\;\;\vdots \;\;\;\;\;\;\;\;\;\;\;\;\;\;\;\;\vdots \;\;\;\;\;\;\;\;\;\;\;\;\;\;\vdots \\ e_{}^{-j2\pi \frac{1}{T}(L-1)}\;\;\;\cdots \;\;e_{}^{-j2\pi \frac{K}{T}(L-1)} \end{array}\right]\;\;$$

where *W* is the weighting matrix. $p[{\left|s\right|}_{}^{2}]$ is the matrix containing the fault characteristic cycle frequency. *K* is the number of samples. *T* represents the failure cycle. The cycle frequency is the frequency related to the signal pulse, which is related to mechanical equipment failures such as bearings.

Formula (25) is the core of CYCBD. The weighting matrix *W* can be calculated by the inverse filter *h* and the observation signal *x*. Then, using the characteristics of generalized Rayleigh entropy, it can be concluded that the optimal inverse filter *h* is equivalent to the maximum eigenvector corresponding to the maximum generalized eigenvalue of the weighted correlation matrix $R_{XWX}$ and the correlation matrix $R_{XX}$ in the Formula (24). Then, the calculation result is converted into Formula (21) and the deconvolution signal corresponds to the maximum second-order cyclostationarity, and the fault frequency can be extracted from the observation signal *x*.

## 3. The Process of Fault Feature Extraction

### 3.1. Parameter Optimization of VMD Based on MPA

In the MPA algorithm optimizing VMD, the selection of the fitness function directly affects the optimization effect. Once the bearing is damaged during operation, it will produce a pulse impact. This method can be used to characterize the irregularity of the time series. The greater the entropy of dispersion, the higher the degree of irregularity.

**Figure 1:**
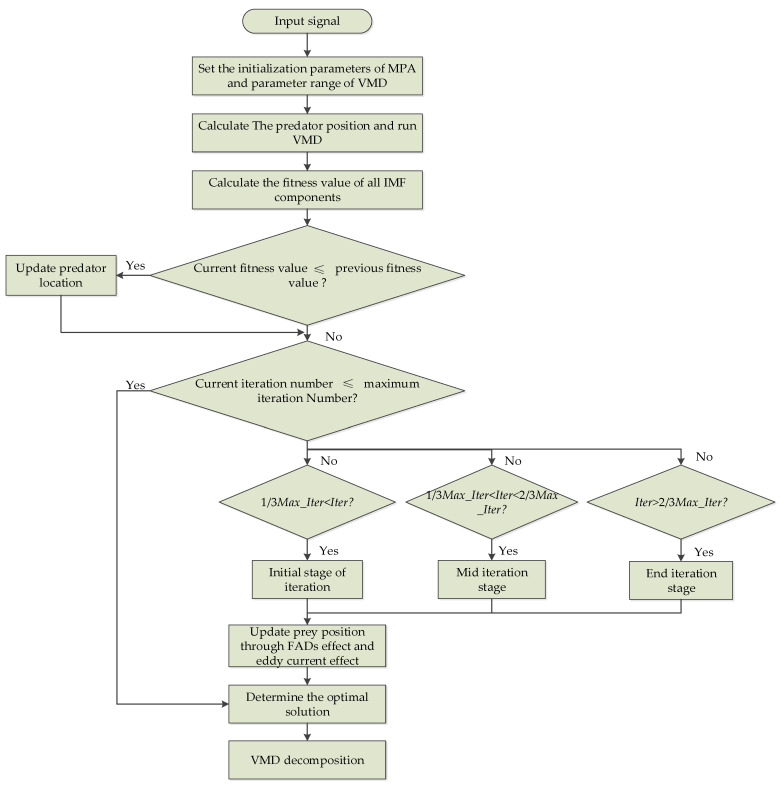
Flowchart of MPA-VMD.

Therefore, the concept of dispersion entropy is used to measure the irregularity of the intrinsic mode function (IMF) time series data obtained by VMD and took the average value of the dispersion entropy of the signal component as the fitness function of MPA. Then, through the global optimization of the mean value of the minimum dispersion entropy, the optimal decomposition level *k* and the penalty factor *α*are obtained. The flow chart of MPA-VMD is shown in Figure 1. The specific steps are as follows:

(1) Initialize the parameters of the MPA, including the population size, maximum iterations, *FADs*, and the parameter range to be optimized for VMD.
(2) Set the fitness function value, then obtain the initial position of the prey and calculate the fitness value.
(3) Iterative optimization is performed from the initial, middle, and late stages of the iteration. Comparing the fitness values in the iteration process can detect whether the optimal fitness value can be found and then update the predator’s position. Then, calculate the fitness of the new location and evaluate the impact of the *FADs* or eddy current effect on the fitness value. Calculate the optimal predator position according to the prey position and behavior and, finally, determine and store the current optimal position.
(4) Judge whether the following relationship is true: iter ≥ Max_iter. If not, continue to repeat steps 2 and 3 to find the optimal solution. Otherwise, the calculation is terminated and the set of optimal parameters is the output.
(5) The optimal parameters obtained from the MPA algorithm optimization are assigned to VMD to build the algorithm model.

### 3.2. IMF Component Selection Criteria

After VMD, the IMF components obtained may have false components or components with a small correlation with the original signal. If the practical signal components are not filtered, the subsequent noise reduction and fault feature extraction will be somewhat disturbed. Therefore, this paper established a combined weight-effective signal component screening rule that combines kurtosis and cross-correlation coefficients.

The cross-correlation coefficient can represent the interdependence between two signal amplitudes. The greater the cross-correlation coefficient between the IMF signal components and the original signal, the stronger the degree of correlation. Kurtosis is an index used to judge the Gaussian performance of vibration signals, which can be used to know whether each IMF signal component carries shock and fault components from the side. Kurtosis reflects the impact of the impact components.

Although kurtosis is sensitive to impact components, proper signals are vulnerable to noise interference. Although the correlation coefficient can measure the correlation between signals, it is easily affected by the total number of samples. By combining the advantages and disadvantages of the above two evaluation indicators, the calculation method based on the combination weight value is as follows:

(29) $$K-C=\log_{2}(1+\phi *Kurtosis+\varphi *Collelation)$$

where ϕ and φ are the weights of kurtosis and the correlation coefficient, respectively, and ϕ+φ=1 is satisfied. In combination with the parameter selection rules in the literature [31], this paper has selected ϕ = 0.4, φ = 0.6 through the experiments.

### 3.3. Implementation Process

**Figure 2:**
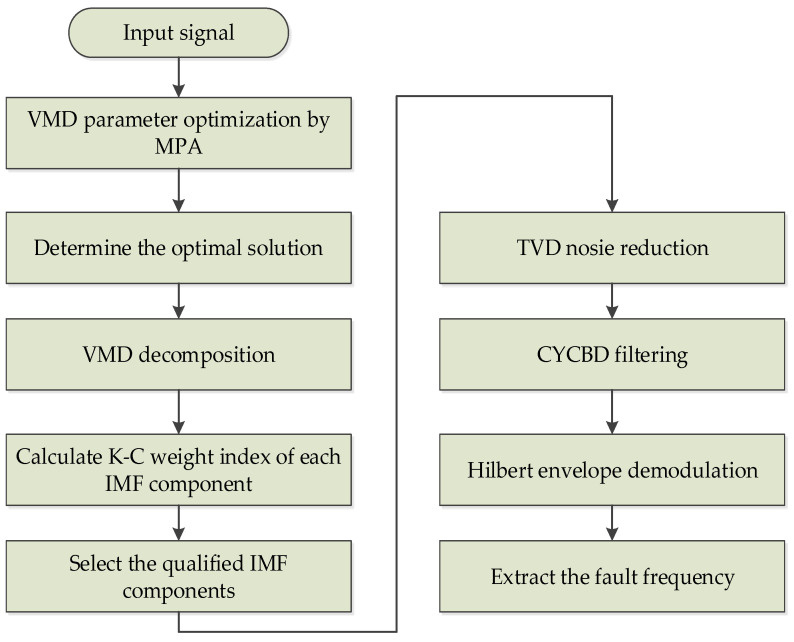
Flow chart of the improved VMD multi-scale dispersion entropy and TVD-CYCBD method.

The implementation process of the proposed method is as follows. The specific implementation flowchart is shown in Figure 2.

(1) Optimize VMD parameters. Initialize the parameters of the MPA algorithm and determine the optimal *k* and *α* by optimizing VMD through MPA.
(2) The best parameters are substituted into the VMD algorithm model to complete the modeling. Then, the VMD is carried out for the collected signals and several signal components are obtained. The K-C index of each IMF is calculated according to the screening criteria based on the K-C combination weight. Then, we can evaluate the quality of the IMF signal components. Finally, select the IMF signal component with the largest K-C value.
(3) Input the filtered IMF signal components into TVD for noise reduction to reduce noise interference in valuable signals.
(4) The fault frequency of the original signal is calculated according to the fault frequency formula and the appropriate cycle frequency is set. Then, the signal is filtered by CYCBD.
(5) The signal filtered by CYCBD is analyzed by Hilbert envelope.

## 4. Simulation Verification

To determine whether the proposed method based on improved VMD multi-scale dispersion entropy and TVD-CYCBD can extract the fault pulse impact component, this research constructs the rolling bearing vibration simulation signal:

(30) $$s(t)=y_{0}e_{}^{-2\pi f_{n}\xi t}\sin(2\pi f_{n}\sqrt{1-\xi_{}^{2}}t)$$

where *y*_0_ is the displacement constant and its value is 5. *f_n_* is the carrier frequency and its value is 3000 Hz. ξ is the damping coefficient and its value is 0.1. *f_s_* is the sampling frequency and its value is 20 KHz. *t* indicates the sampling time. *T* = 0.01 s. The sampling points are *N* = 4096. The fault frequency is *f*_0_ = 100 Hz.

**Figure 3:**
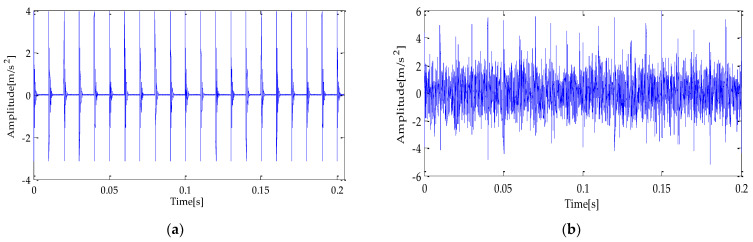
(**a**) Simulated fault signal; (**b**) simulated fault signal with noise.

To simulate the bearing fault according to the actual situation, this experiment adds noise to the simulation signal *s*(*t*), and its signal-to-noise ratio (SNR) is −5 dB. The waveform of the simulation signal is shown in Figure 3. The time and frequency domain diagram of the simulation signal after adding noise are shown in Figure 3a,b.

**Figure 4:**
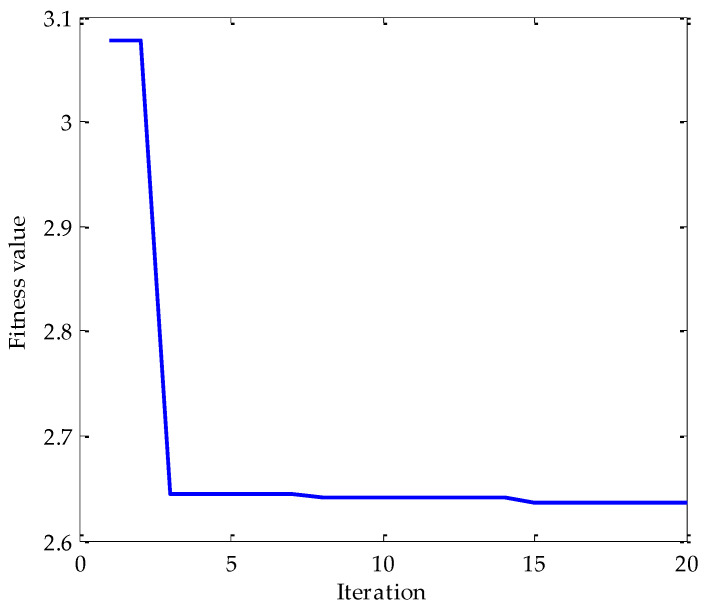
The change in fitness curve.

**Figure 5:**
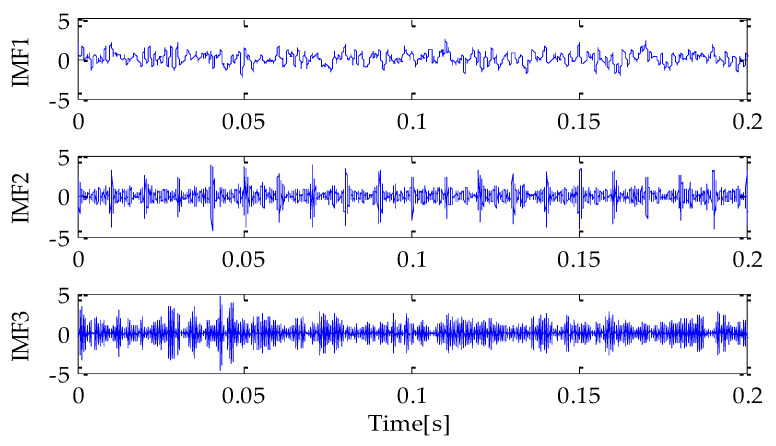
VMD result.

**Figure 6:**
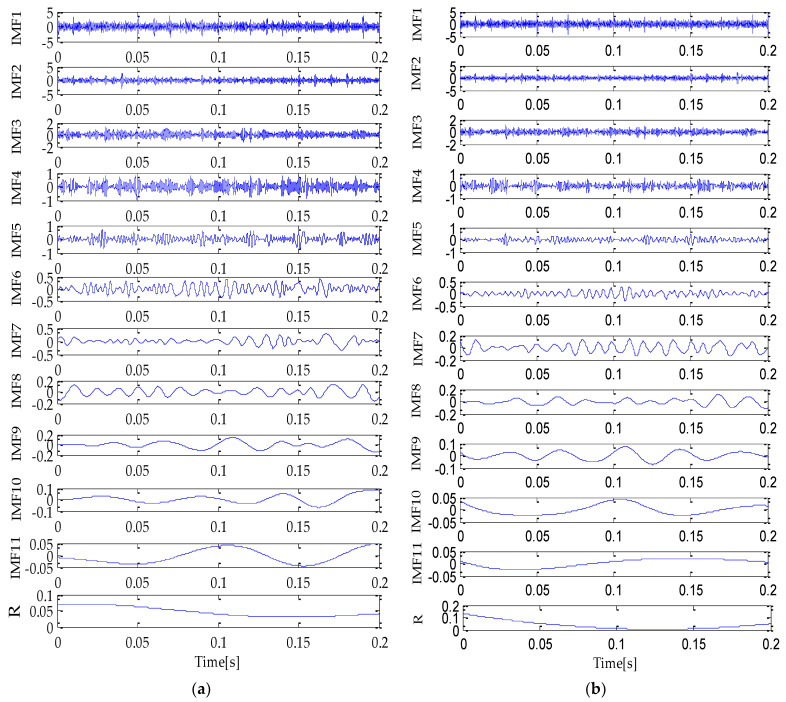
EMD (**a**) and EEMD (**b**) decomposition result.

Firstly, the MPA optimization algorithm is used to optimize the parameters in VMD. The parameter search range in VMD is as follows: the *k* ∊ [3, 15] and the *α* ∊ [100, 5000]. In the MPA algorithm, the population size is 10 and the *FADs* are 0.2. Meanwhile, the maximum iteration number is 20. In the process of optimization, the fitness function adopted is the average value of dispersion entropy. After parameter optimization, the fitness curve of the MPA obtained is shown in Figure 4. According to the results shown in Figure 4, when the fitness value is optimal, the corresponding *k* and *α* are 3 and 3348, respectively. Therefore, the above parameters are substituted into the VMD for modeling and then VMD is performed on the simulation signal added with noise. To compare and analyze the effects of the MPA-VMD method and other methods, this research also conducted experiments based on the EMD and EEMD methods. The decomposition results obtained based on MPA-VMD, EMD, and EEMD are shown in Figure 5 and Figure 6.

Compared with Figure 5 and Figure 6, it is shown that the number of signal components obtained based on MPA-VMD is the least, and the modal components have a good separability and weak modal aliasing. More signal components are obtained based on the EMD and EEMD methods: 11 signal components and 1 residual component. Meanwhile, it can be seen that some signal components have severe mode aliasing and many false components are generated. Next, the kurtosis and correlation values of the signal components decomposed by the MPA-VMD, EMD, and EEMD methods will be calculated. Each signal component’s K-C combined weight index will be calculated according to the calculation formula of the combined weight proposed in this paper. The calculation results based on MPA-VMD, EMD, and EEMD are shown in Table 1, Table 2 and Table 3.

By comparing the K-C combined weight index of each signal component in Table 1, it is shown that the result value of IMF1 obtained based on the MPA-VMD method is the largest. The correlation and kurtosis values of this signal component are the largest compared with other signal components. Therefore, IMF1 is selected as the optimal signal component for the reduction in signal noise. Comparing the K-C combined weight index of each signal component in Table 2 shows that the result value of IMF2 obtained based on the EMD method is the largest. Therefore, IMF2 is selected as the optimal signal component for the reduction in the signal noise. Comparing the K-C combined weight index of each signal component in Table 3 shows that the result value of IMF2 obtained based on the EEMD method is the largest. Therefore, according to the comparison of the computer values, the IMF2 will be used as the optimal signal component for the reduction in the signal noise. Furthermore, it can also be seen from Table 1, Table 2 and Table 3 that using one of the cross-correlation coefficients and kurtosis values to filter will cause a specific interference in the filtering of the signal components.

**Figure 7:**
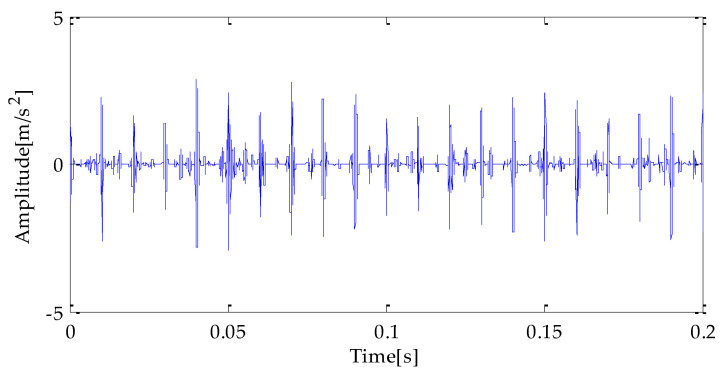
MPA-VMD-TVD noise reduction results.

**Figure 8:**
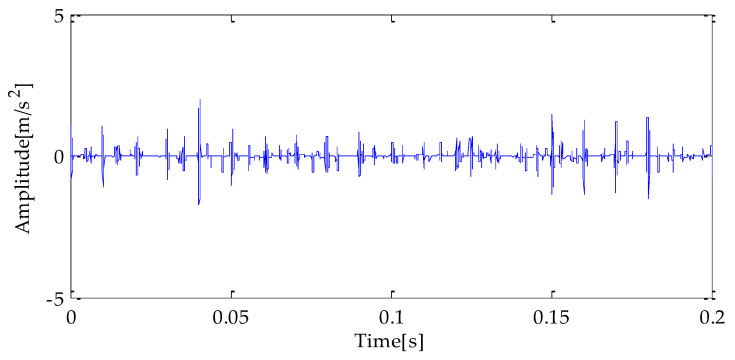
EMD-TVD noise reduction results.

**Figure 9:**
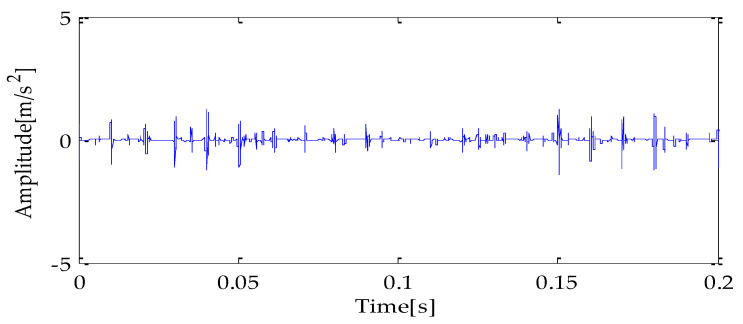
EEMD-TVD noise reduction results.

Figure 7, Figure 8 and Figure 9 are the time-domain waveforms of the signal components filtered based on MPA-VMD, EMD, and EEMD methods after noise reduction. Through analysis, the following conclusions can be drawn: after the TVD noise reduction, the noise interference has been reduced to varying degrees. Several evaluation indexes are introduced in this study to compare the noise reduction effects. They are SNR, RMSE, and MAE. Calculating the above index values shows the results in Table 4.

The data shown in Table 4 are the results of the three methods. It shows that the SNR based on the MPA-VMD-TVD method is 4.225, the correlation coefficient is 0.792, the RMSE is 0.357, and the MAE is 0.179. Compared with EMD-TVD and EEMD-TVD, the SNR is higher and the RMSE is smaller. Therefore, according to the above results, we can come to the conclusion that the method proposed in this paper has a better noise reduction effect.

**Figure 10:**
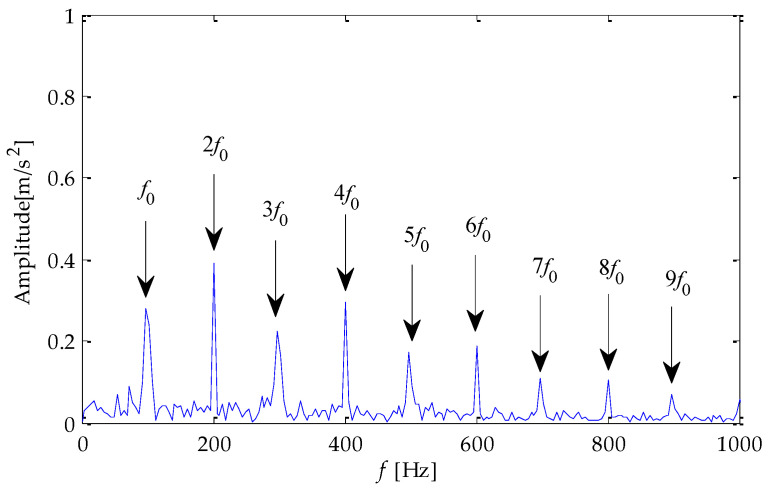
Envelope spectrum based on MPA-VMD-TVD.

**Figure 11:**
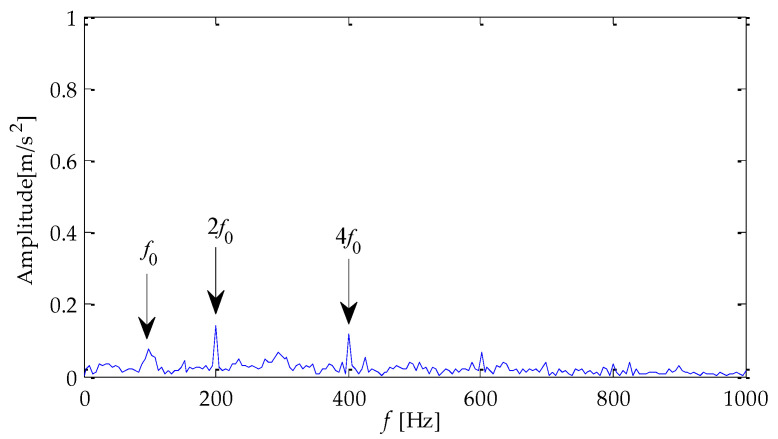
Envelope spectrum based on EMD-TVD.

**Figure 12:**
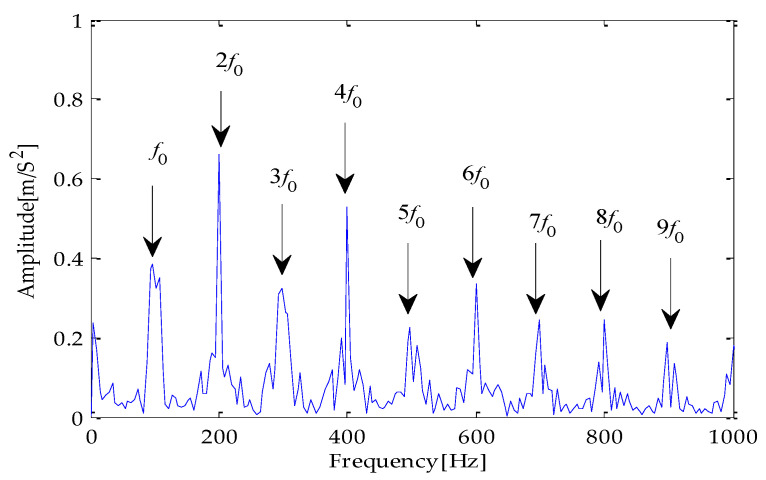
Envelope spectrum based on EEMD-TVD.

Next, envelope spectrum analysis is performed on the noise-reduced signals using the above three methods. The envelope spectrum obtained by the hilbert transformation is shown in Figure 10, Figure 11 and Figure 12. Through the analysis of the above three results, it can be seen that the fault characteristic frequency and its multiple frequency can appear in the envelope spectrum obtained based on the EMD-TVD method. However, the amplitude of the fault frequency is relatively low, which brings some interference to the extraction of the fault characteristics. Meanwhile, although the characteristic frequency of the fault and its double frequency, three-time frequency, four-time frequency, and other components can be seen from Figure 10, due to an insufficient signal noise reduction, the interference of other irrelevant frequencies exists near the peak value of the multiple fault frequencies. From the envelope spectrum obtained on the basis of MPA-VMD-TVD, the fault characteristic frequency and its double frequency, three-time frequency, four-time frequency, and other components are clearly displayed. Meanwhile, the outside interference near the fault frequency is significantly reduced.

**Figure 13:**
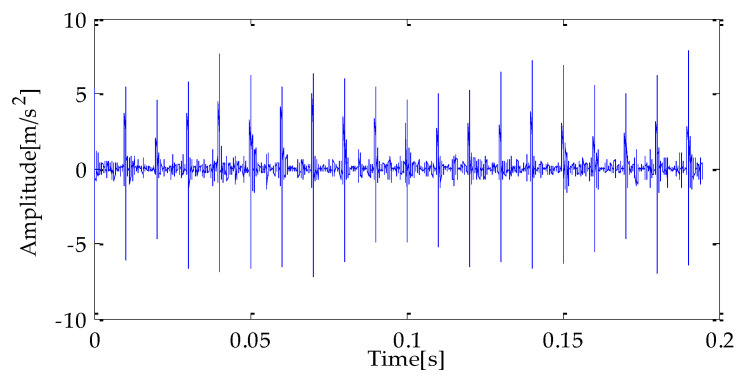
CYCBD filter signal based on MPA-VMD-TVD.

**Figure 14:**
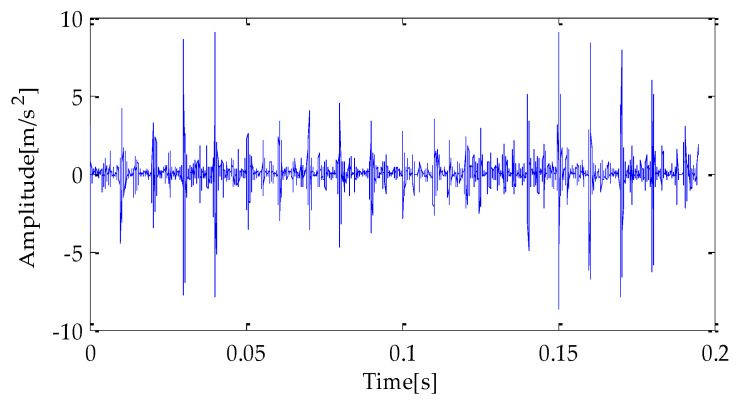
CYCBD filter signal based on EMD-TVD.

**Figure 15:**
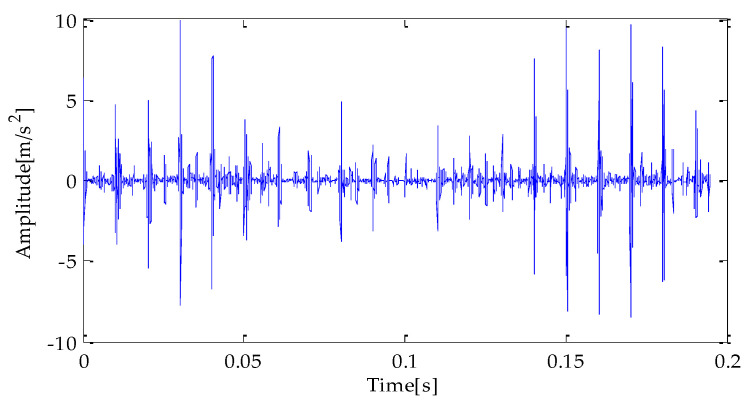
CYCBD filter signal based on EEMD-TVD.

**Figure 16:**
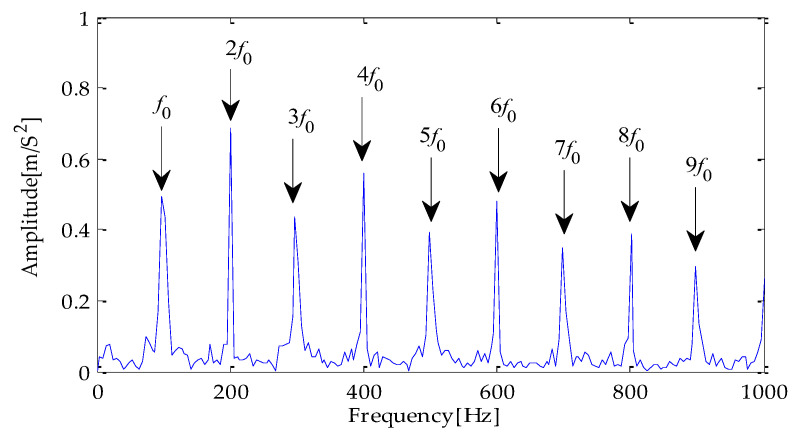
Envelope spectrum of CYCBD filter signal based on MPA-VMD-TVD.

**Figure 17:**
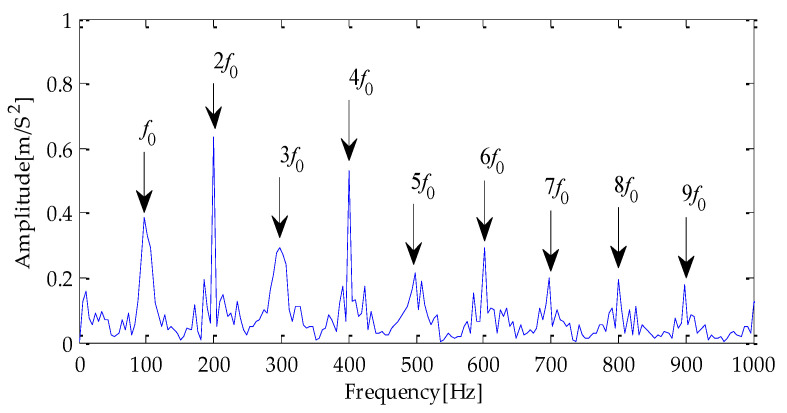
Envelope spectrum of CYCBD filter signal based on EMD-TVD.

**Figure 18:**
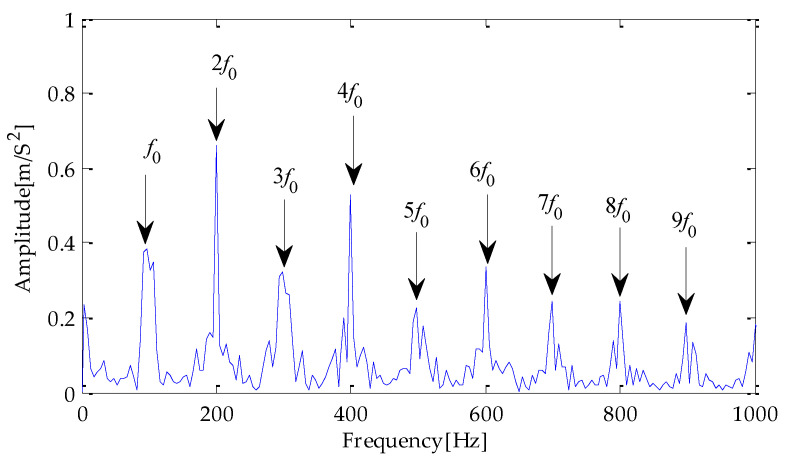
Envelope spectrum of CYCBD filter signal based on EEMD-TVD.

Finally, the above three signals after TVD noise reduction are inputted to the CYCBD filter for further filtering to enhance the signal’s periodic impact characteristics. In this algorithm, the cyclic frequency set is set to [100,200, …,1000]. Then, the signal filtered by the CYCBD is analyzed in the envelope. The time domain diagrams of the three signals filtered by the CYCBD filter are shown in Figure 13, Figure 14 and Figure 15, and the generated envelope spectrum is shown in Figure 16, Figure 17 and Figure 18. It can be seen from Figure 13, Figure 14 and Figure 15 that after filtering, the periodic impact components in the time domain diagrams based on MPA-VMD-TVD, EMD-TVD, and EEMD-TVD methods have been greatly improved. Through the comparative analysis of Figure 16, Figure 17 and Figure 18, it is shown that the characteristic frequency of the faultand its double to nine times of the fault frequency can be extracted by using the above three methods. At the same time, in general, the amplitude of the fault frequency and multiple frequencies of the fault frequency obtained based on the proposed method are the highest, and there is no outside interference near the fault frequency. However, there are still different degrees of outside interference near the fault frequency obtained by the other two methods. Therefore, the proposed method achieved better results.

## 5. Experimental Verification

**Figure 19:**
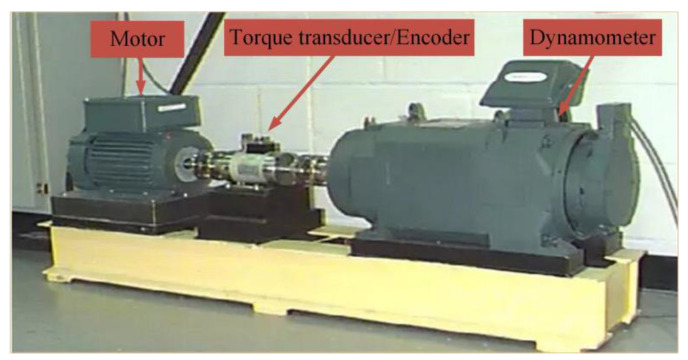
Rolling bearing fault acquisition equipment.

The experimental data used in this study are from the public data set of CWRU [32]. The fault acquisition equipment is shown in Figure 19 [33] and the bearing structure coefficient is shown in Table 5. The motor speed is 1797 rpm. The sampling frequency is 12 KHz. The model used at the drive end is 6205-2RSJEMSKF, the motor speed is 1797 rpm, and the sampling frequency is 12 KHz.

### 5.1. Analysis of Inner Ring Vibration Signal

**Figure 20:**
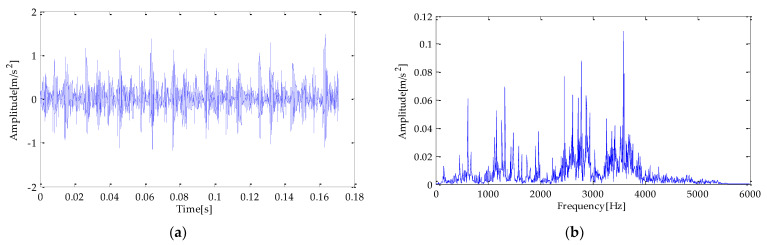
(**a**) original fault signal; (**b**) frequency domain diagram.

**Figure 21:**
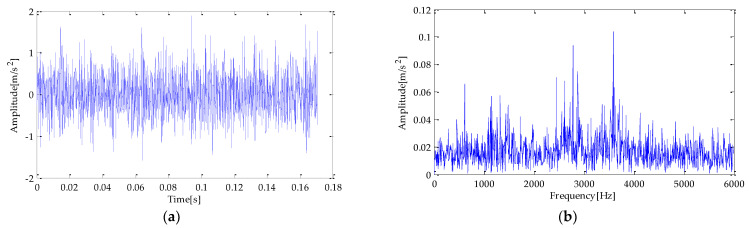
(**a**) original fault signal with noise; (**b**) frequency domain diagram.

The time and frequency domain waveforms of the experimental signal are shown in Figure 20a,b. To test the performance of the method in terms of the anti-noise interference, this experiment adds noise to the original signal, and its SNR is −2dB. The signal’s time and frequency domain diagram added with noise are shown in Figure 21a,b.

**Figure 22:**
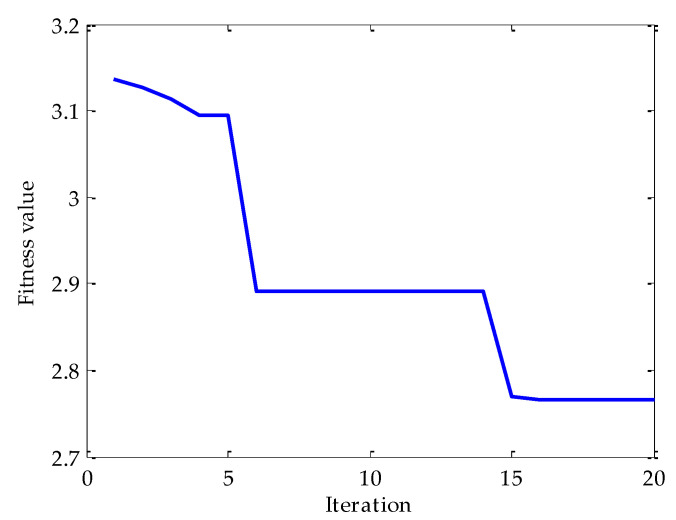
The change in fitness curve.

**Figure 23:**
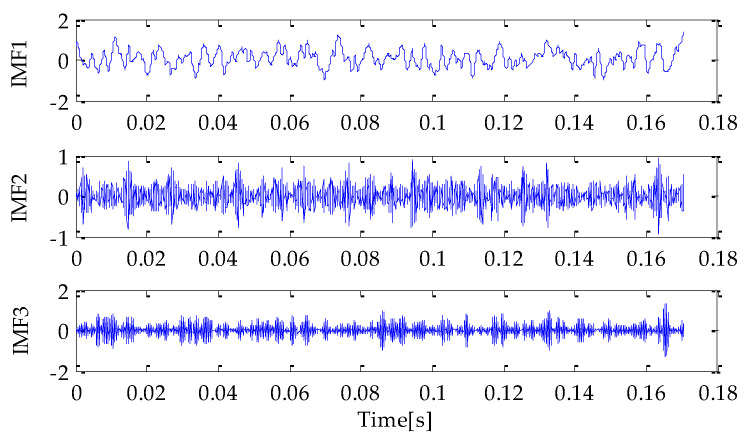
VMD result.

Firstly, the MPA is used to optimize the decomposition level *k* and the penalty factor *α* according to the VMD parameter optimization process proposed in this paper. The fitness function used in MPA optimization is the average value of dispersion entropy. The parameter search range in VMD is as follows: *k* ∊ [3, 15] and *α* ∊ [100, 3000]. In the MPA algorithm, the population size is 10. Meanwhile, the maximum iteration number is 20 andthe *FADs* are 0.2. After parameter optimization, the fitness curve of the obtained MPA is shown in Figure 22. According to the results shown in Figure 22, when the fitness value is optimal, the decomposition level *k* and penalty factor *α* are 3 and 1916, respectively. Therefore, *k* = 3 and *α* = 1916 are taken as the optimal values. Then, the above two parameters are substituted into the VMD algorithm for modeling, and VMD decomposes the bearing inner race fault signal after adding noise. The decomposition result is shown in Figure 23.

Next, the kurtosis and correlation values of the signal components decomposed by the above MPA-VMD method are calculated and the K-C index of each IMF are calculated according to the formula of the combined weights. The calculation results of the signal components are shown in Table 6. Comparing the K-C index of each signal component in Table 6 indicates that the K-C index of the IMF2 component obtained based on the MPA-VMD method is the largest. Therefore, IMF2 is selected as the optimal signal component for the next TVD noise reduction operation.

**Figure 24:**
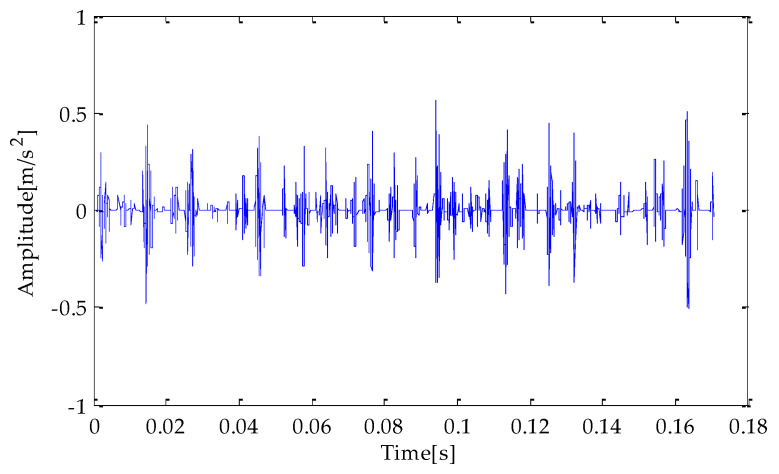
MPA-VMD-TVD noise reduction results.

**Figure 25:**
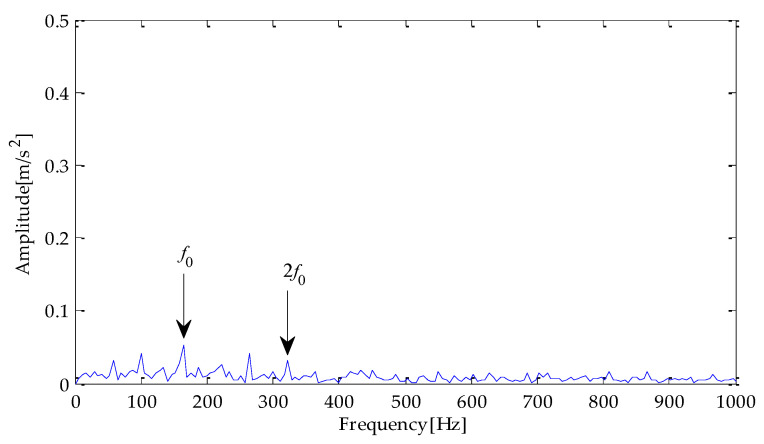
Envelope spectrum based on MPA-VMD-TVD.

Figure 24 is the noise reduction waveform of the optimal signal components filtered based on the MPA-VMD method. It shows that the noise interference is reduced to a certain extent by the TVD method. Then, the signal after noise reduction is analyzed by the Hilbert envelope. The following conclusions are drawn from the envelope spectrum in Figure 25: the fault feature frequency and its double frequency appear in the figure. Still, their amplitude is low, complicating the fault characteristics’ effective extraction. Next, the noise-reduced signal is input into the CYCBD filter. In this algorithm, the cyclic frequency set is set to [162.19, 324.38, 486.57, …,1135.33].

**Figure 26:**
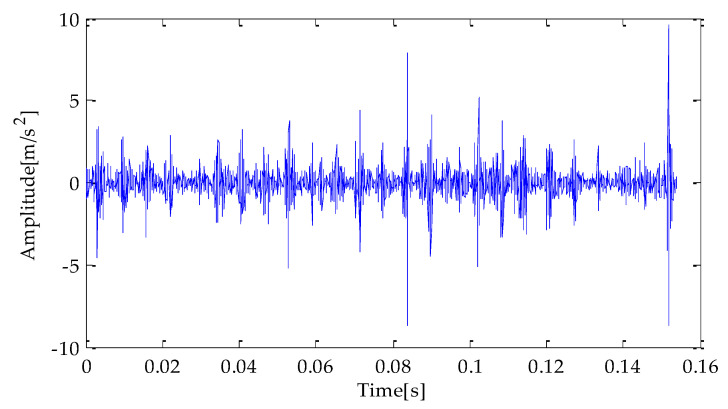
CYCBD filter signal based on MPA-VMD-TVD.

**Figure 27:**
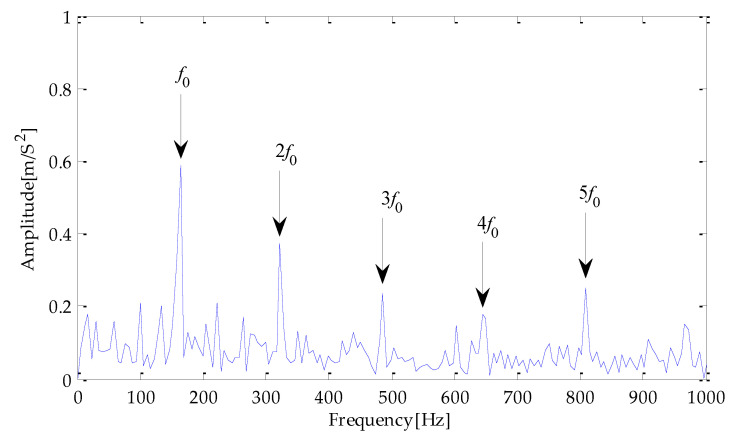
Envelope spectrum of CYCBD filter signal based on MPA-VMD-TVD.

The signal filtered by the CYCBD filter is shown in Figure 26 and the generated envelope spectrum is shown in Figure 27. Observing the waveform shows that the periodic impact component has been significantly enhanced. It is shown in Figure 27 that the frequency characteristic of the fault and its double frequency, three-times frequency, four-times frequency, and five-times frequency components have appeared, and the amplitude is exceptionally high. Therefore, it can be determined that the bearing has inner race failure.

### 5.2. Analysis of Outer Ring Vibration Signal

**Figure 28:**
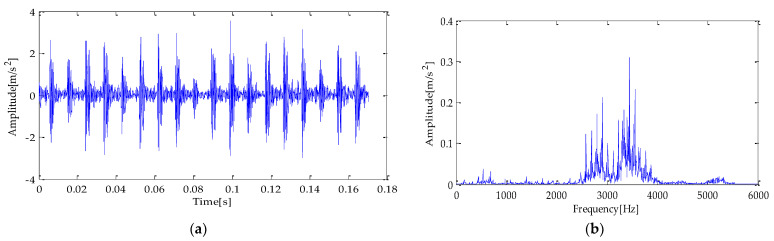
(**a**) original fault signal; (**b**) frequency domain diagram.

**Figure 29:**
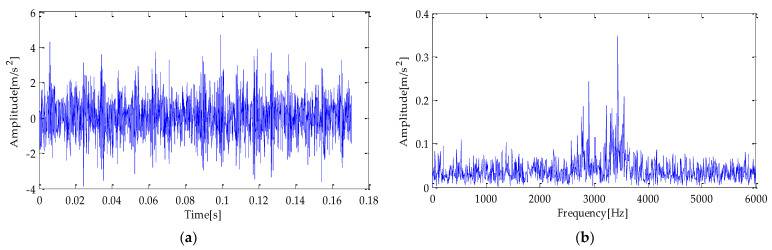
(**a**) original fault signal with noise; (**b**) frequency domain diagram.

The time and frequency domain waveforms of the experimental signal are shown in Figure 28a and Figure 29b. Similarly, to verify the method’s performance in anti-noise interference, the experiment adds noise to the original signal, and its SNR is−2dB. The time and frequency diagram of the signal after adding noise are shown in Figure 29a,b.

**Figure 30:**
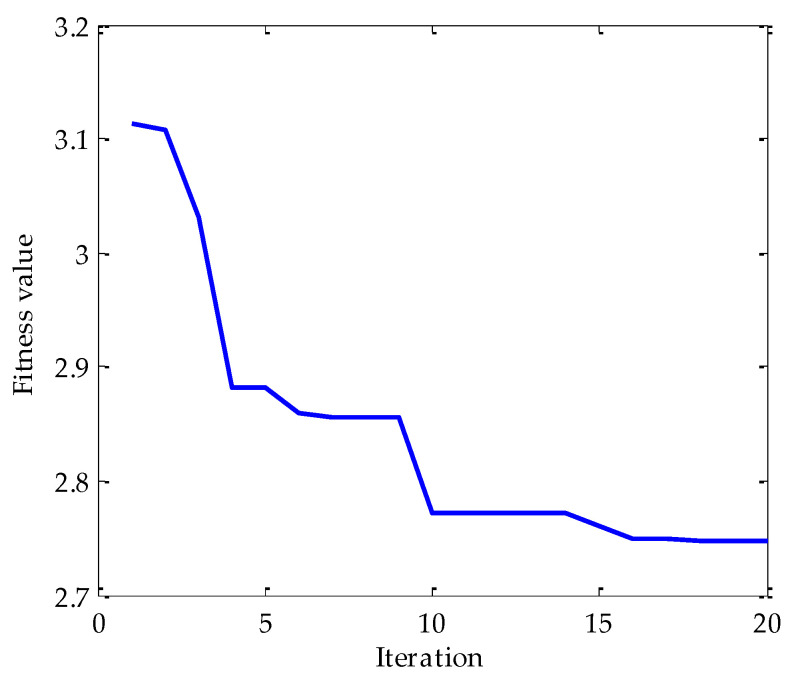
The change in fitness curve.

**Figure 31:**
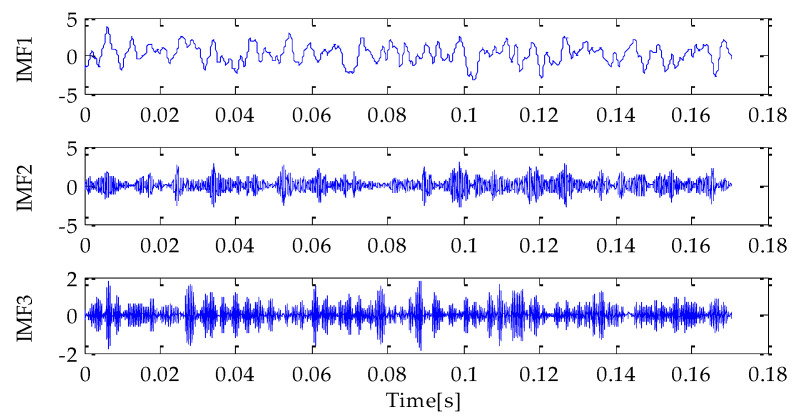
VMD result.

Next, the MPA optimization algorithm is used to optimize the *k* and *α* in VMD. Similarly, the parameter search range in VMD is as follows: the decomposition level *k* ∊ [3, 15] and the penalty factor *α* ∊ [100, 3000]. In the MPA algorithm, the population size is 10, the maximum iteration is 20, and the *FADs* are 0.2. After parameter optimization, the fitness curve of the obtained MPA is shown in Figure 30. It is shown from the change in the results of the fitness curve that when the fitness value is optimal, the corresponding optimal *k* and *α* are 3 and 2541, respectively. Therefore, the above two parameters are substituted into the VMD for modeling, and then VMD is carried out for the simulation experiment signal added with excessive noise. The decomposition result is shown in Figure 31.

Next, the kurtosis and correlation values of the signal components decomposed by the MPA-VMD method are calculated, and the K-C combination weight calculation formula calculates the K-C index of each IMF. The calculation results of the signal components are shown in Table 7. By comparing the K-C index of each signal component in Table 7, it is shown that the K-C index of the IMF2 component obtained based on the MPA-VMD method is the largest. Therefore, the IMF2 is selected as the optimal signal component for the next TVD noise reduction operation.

**Figure 32:**
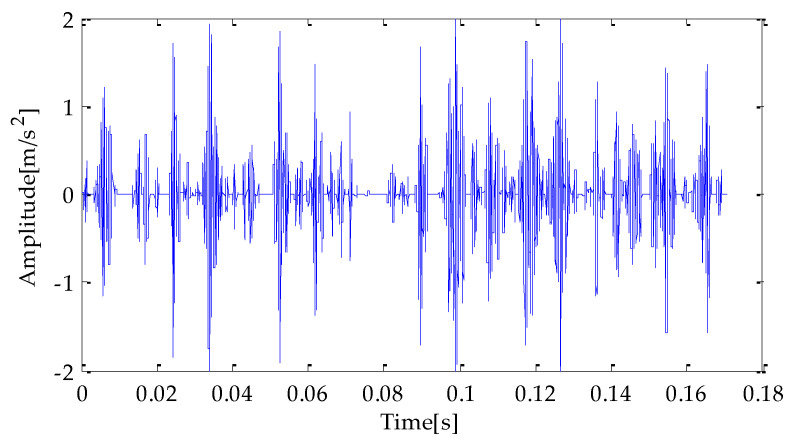
MPA-VMD-TVD noise reduction results.

**Figure 33:**
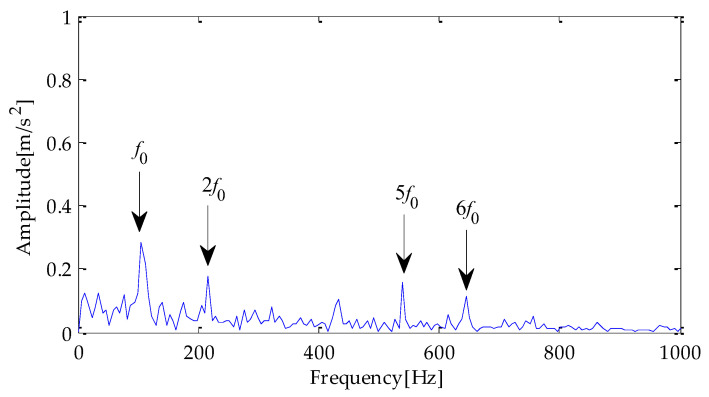
Envelope spectrum based on MPA-VMD-TVD.

Figure 32 shows the waveform after the noise reduction of the optimal signal components filtered based on the MPA-VMD method. Noise interference is shown to be significantly reduced by the TVD method. Then, a Hilbert envelope analysis is performed on the noise-reduced signal and the results are shown in Figure 33. It can be seen from Figure 33 that the fault characteristic frequency, double frequency, five-times frequency, and six-times frequency components appear in the envelope spectrum. However, their amplitude is low, and some irrelevant interference components appear around them, which makes it difficult to extract the fault features effectively. Next, the noise-reduced signal is input into the CYCBD filter. In this algorithm, the cyclic frequency set is set to [107.36, 214.72, 322.08, …, 966.24].

**Figure 34:**
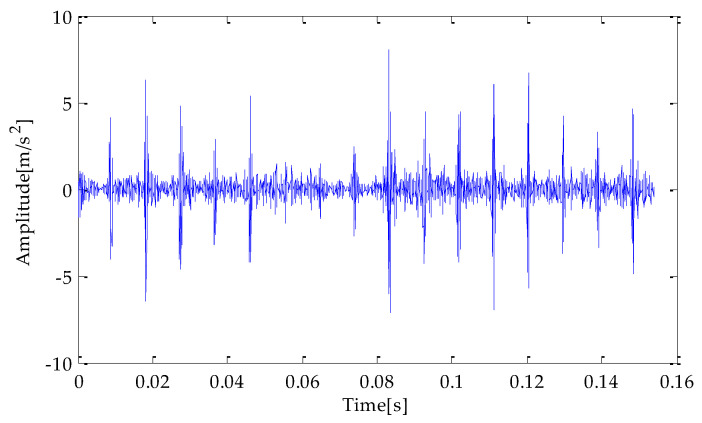
CYCBD filter signal based on MPA-VMD-TVD.

**Figure 35:**
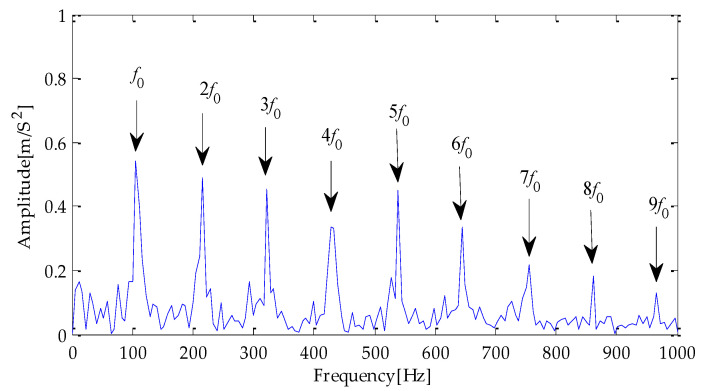
Envelope spectrum of CYCBD filter signal based on MPA-VMD-TVD.

The signal filtered by the CYCBD filter is shown in Figure 34 and the envelope spectrum generated is shown in Figure 35. It is shown that Figure 34 that the periodic impact component has been significantly improved. At the same time, the fault characteristic frequency and its second- to nine-times frequency components have appeared and the amplitude is exceptionally high. Therefore, it can be determined that the bearing has outer ring failure.

## 6. Conclusions

In this paper, the method combining improved VMD multi-scale dispersion entropy and TVD-CYCBD is used to research the fault feature extraction of rolling bearing signals under noise interference. The conclusions are as follows.

(1) The MPA is used to optimize the decomposition level and penalty factor in the VMD algorithm to find the optimal parameter combination. It can avoid over-decomposition and under-decomposition problems caused by the traditional VMD algorithm and realize the proper decomposition and the reconstruction of signals. This method overcomes the difficulties of mode aliasing and the end effect caused by impact components and noise interference in EMD and EEMD methods.
(2) The K-C index is constructed by balancing the advantages and disadvantages of kurtosis and the cross-correlation coefficient, which solves the problems of identifying the optimal signal components. It effectively removes the component signals with a weak correlation with the original signal. Second, the TVD method reduces the noise of the selected optimal component. It effectively reduces the noise interference. Compared with the noise reduction results obtained based on EMD and EEMD methods, the waveform of the signal received by the proposed noise reduction method is more similar to the original signal. It is also optimal in comparing the noise reduction evaluation index results.
(3) The fault impact component can be better highlighted by CYCBD filtering on the signal after TVD denoising. In the simulation signal experiment and the experimental verification of the CWRU data, the envelope spectrum generated by the proposed method can successfully extract the fault frequency and its multiple frequencies. At the same time, the result of fault feature extraction is better than that based on EMD and EEMD, which shows the effectiveness of the proposed method.

## Figures and Tables

**Table 1:** K-Cindex (MPA-VMD).

	IMF1	IMF2	IMF3
*C*(*t*)	0.346	0.618	0.281
*Q*(*t*)	3.055	6.915	3.193
K-C	1.281	2.049	1.290

**Table 2:** K-C index (EMD).

	IMF1	IMF2	IMF3	IMF4	IMF5	IMF6
*C*(*t*)	0.740	0.516	0.281	0.202	0.155	0.103
*Q*(*t*)	3.355	4.270	3.107	3.012	3.216	2.425
K-C	1.478	1.593	1.270	1.218	1.250	1.023
	**IMF7**	**IMF8**	**IMF9**	**IMF10**	**IMF11**	**R**
*C*(*t*)	0.064	0.022	0.024	0.005	0.006	0.006
*Q*(*t*)	3.831	2.479	2.578	2.674	1.702	1.634
K-C	1.362	1.003	1.033	1.052	0.752	0.729

**Table 3:** K-C index (EEMD).

	IMF1	IMF2	IMF3	IMF4	IMF5	IMF6
*C*(*t*)	0.794	0.578	0.329	0.250	0.179	0.113
*Q*(*t*)	3.671	4.499	3.220	3.376	3.217	2.826
K-C	1.558	1.654	1.314	1.322	1.260	1.136
	**IMF7**	**IMF8**	**IMF9**	**IMF10**	**IMF11**	**R**
*C*(*t*)	0.079	0.062	0.029	0.018	0.004	0.012
*Q*(*t*)	2.130	2.810	2.308	2.090	1.687	3.365
K-C	0.925	1.112	0.956	0.885	0.746	1.235

**Table 4:** Noise reduction effect analysis.

Index	EMD-TVD	EEMD-TVD	MPA-VMD-TVD
SNR	1.120	1.023	4.225
Correlation coefficient	0.481	0.487	0.792
RMSE	0.510	0.516	0.357
MAE	0.197	0.179	0.179

**Table 5:** The bearing structure factor.

Rolling Element Number	Inner Diameter	Outer Diameter	Contact Angle	Pitch Circle Diameter *D*
9	0.9843	2.0472	0°	1.5327

**Table 6:** K-C index (VMD).

	IMF1	IMF2	IMF3
*C*(*t*)	0.269	0.651	0.300
*Q*(*t*)	2.749	3.837	3.150
K-C	1.261	1.926	1.440

**Table 7:** K-C index (VMD).

	IMF1	IMF2	IMF3
*C*(*t*)	0.269	0.651	0.300
*Q*(*t*)	2.749	3.837	3.150
K-C	1.261	1.926	1.440

## Data Availability

Not applicable.
